# Supplementary material for: DNA-Based Electrodes and Computational Approaches on the Intercalation Study of Antitumoral Drugs
Source: Molecules. 2021 Dec 16;26(24):7623. doi: 10.3390/molecules26247623 (PMC8709249; doi:10.3390/molecules26247623)
Supplement: Supplementary file 1 [file molecules-26-07623-s001.zip › molecules-1431308-supplementary.pdf]

SUPPORT INFORMATION

Table S1. Docking scores obtained with B-DNA dodecamer and DNA hexamer structures.

Drug	B-DNA dodecamer		DNA hexamer	
	Glide Emodel	GlideScore (kcal/mol)	Glide Emodel	GlideScore (kcal/mol)
Doxorubicin	-66.10	-8.83	-79.26	-10.33
Daunorubicin	-61.83	-6.67	-73.38	-8.86
Idarubicin	-79.16	-7.19	-79.47	-10.22
Dacarbazine	-42.29	-1.84	-44.14	-3.81
Mitoxantrone	-112.43	-10.89	-100.57	-11.88
Methotrexate	-65.14	-0.99	-45.68	-8.82
